# Editorial: Next generation solutions for efficient management of phytopathogens

**DOI:** 10.3389/fmicb.2024.1390670

**Published:** 2024-03-13

**Authors:** Nilanjan Chakraborty, Ugo De Corato, Chetan Keswani, Estibaliz Sansinenea

**Affiliations:** ^1^Department of Botany, Scottish Church College, University of Calcutta, Kolkata, India; ^2^Italian National Agency for New Technologies, Energy and Sustainable Economic Development (ENEA), Rome, Italy; ^3^Academy of Biology and Biotechnology, Southern Federal University, Rostov-on-Don, Russia; ^4^Facultad de Ciencias Químicas, Benemérita Universidad Autónoma de Puebla, Puebla, Mexico

**Keywords:** crops, phytopathogens, agriculture, biocontrol agents, disease management

Ensuring food security for a rapidly growing human population requires colossal efforts to increase the yield of crops, especially under the threat of climate change. The currently employed methodologies in agriculture have not been able to significantly prevent and diminish crop losses due to plant diseases and this has been a major concern during the last decade. Crops are affected by a wide range of pathogens, including bacteria, fungi, and viruses, causing infections that are responsible for significant yield losses in several economically important crop species, posing a challenge to global food security and the agricultural industry.

Scientists have made great efforts to explore possible solutions to manage economically important phytopathogens. One of the most employed methods consists of using chemical pesticides or fungicides. However, there have been several concerns regarding the health and environmental effects of pesticides, as well as the risk of potential phenomena related to the development of resistant pathogenic strains to fungicides. Therefore, other strategies have had to be taken, such as biological control agents (Pandit et al., 2022).

In this Research Topic, the editors aimed to offer a selection of six articles that address different strategies for a broad overview of modern and innovative plant protection.

Exploring the search for a new chemical fungicide, Tetz et al. focused their research on studying the *in-vitro* antifungal activity of a novel synthetic polymer M451 (1,6-diamino hexane derivative) against a collection of phytopathogenic fungi composed of six species of *Fusarium, Blumeria graminis, Claviceps purpurea, Alternaria alternata, Phytophthora infestans*, and *Rhizoctonia solani*. The most encouraging antifungal effects were observed on *Fusarium oxysporum*.

Other strategies include genetic engineering, which has facilitated the production of genetically modified crops for years. Recently, gene-editing techniques, such as CRISPR/Cas9, have been successfully implemented to enhance disease resistance in several important crops. In this issue, there are two articles that use this technology. Carreras-Villaseñor et al. utilized a CRISPR/cas9-edited strain in FspTF transcription factor, which is homologous to Bqt4 in *Fusarium* sp. and associated with the ambrosia beetle *Xylosandrus morigerus*, to evaluate the role in the growth and pathogenesis of the fungus. Understanding the pathogenesis of this fungus could prevent and mitigate plant diseases. The second article is a review, where Rupawate et al. revised the use of CRISPR/Cas9 technology to modulate gut symbionts. The main idea is that the manipulation of the gut symbionts alters their growth, thereby increasing insect mortality. This can lead to controlling the pests that attack important crops. Both articles are focused on understanding the enemy to combat it.

Climate change amplifies plant diseases by changing the range and behavior of crop pests and pathogens. Salunkhe et al. reviewed the negative effects caused by waterlogging and anthracnose-twister disease in onion crops. Furthermore, they revised the possible strategies that could help with the negative impacts of the combined stress effects of concurrent waterlogging and anthracnose-twister disease.

Plants are in constant collaborative and competitive community with several microorganisms, which is crucial for crop disease and adversity resistance, growth, and development. Zhang et al., in their article, explained how changing the cultivation media of crops can change the microbial community, improving the growth of the tomato plant and increasing their positive effect on tomato crop in greenhouse cultivation.

There are some bacteria that also act as phytopathogens. This is the case with the last article topic. Jain et al. studied how to decrease the bacterial growth of *Xanthomonas oryzae* pv *oryzae*, which causes leaf blight on rice. The authors described the isolation of phages against the rice bacterial pathogen *Xanthomonas oryzae* pv. *oryzae*, and whole-genome sequencing of potential and novel phage vB_XooS_NR08 and its efficacy to control bacterial blight disease in rice.

This selection of articles describes a range of different strategies that can be applied to control phytopathogens on several crops under different environmental conditions in various cropping systems. Considering the recent developments in sustainable crop protection and production, we believe that these articles will be interesting for researchers and farmers for the reliability of the results obtained.

## Author contributions

NC: Writing – original draft, Writing – review & editing. UD: Writing – original draft, Writing – review & editing. CK: Writing – original draft, Writing – review & editing. ES: Writing – original draft, Writing – review & editing.
